# XpertTrack: Precision Autonomous Measuring Device Developed for Real Time Shipments Tracker

**DOI:** 10.3390/s16030355

**Published:** 2016-03-10

**Authors:** Liviu Viman, Mihai Daraban, Raul Fizesan, Mircea Iuonas

**Affiliations:** 1 Applied Electronics Department, Faculty of Electronics, Telecommunications and Information Technology, Technical University of Cluj Napoca, George Baritiu 26-28, Cluj Napoca 400114, Romania; Mihai.Daraban@ael.utcluj.ro (M.D.); Raul.Fizesan@ael.utcluj.ro (R.F.); 2 Information Technology in Electronics Research and Development Center, Faculty of Electronics, Telecommunications and Information Technology, Technical University of Cluj Napoca, George Baritiu 26-28, Cluj Napoca 400114, Romania; 3 Lives International 3, Rue Jules Guesde, Levallois Perret, Paris 92300, France; mircea.iuonas@lives-international.com

**Keywords:** real time tracker, remote monitoring of shipments, wireless networks, acceleration and temperature sensors, low power devices, Round Robin

## Abstract

This paper proposes a software and hardware solution for real time condition monitoring applications. The proposed device, called XpertTrack, exchanges data through the GPRS protocol over a GSM network and monitories temperature and vibrations of critical merchandise during commercial shipments anywhere on the globe. Another feature of this real time tracker is to provide GPS and GSM positioning with a precision of 10 m or less. In order to interpret the condition of the merchandise, the data acquisition, analysis and visualization are done with 0.1 °C accuracy for the temperature sensor, and 10 levels of shock sensitivity for the acceleration sensor. In addition to this, the architecture allows increasing the number and the types of sensors, so that companies can use this flexible solution to monitor a large percentage of their fleet.

## 1. Introduction

The need for real time information is growing continuously; therefore the persons who have the information have the power of action. Real time data are very important in commercial shipping. Merchandise (e.g., frozen foods, pharmaceutical or biotechnological products) are susceptible to abuse or contamination during transportation and storage [1,2]. The end customers also expect the delivery at a specific time and in specific conditions [3,4,5,6]. Using XpertTrack, all these events can be monitored with a powerful solution, by knowing at any time the temperature or other parameters of the merchandise. 

The device is capable of measuring temperature (inside the cargo), acceleration, and GPS and GSM positioning. The acquired data are sent to a server through the General Packet Radio Service (GPRS). The server runs dedicated software, which processes and saves the acquired data. In the case of losing the GSM signal, the device is also capable of processing and storing the acquired data from the sensors. The device is designed to measure temperature and acceleration, so that shocks and/or free fall can be detected. However, the architecture can be extended so that further measurements can be done. Another feature developed is the possibility to access and configure the device through wireless communication using an Access Point (AP), which also serves as a client backup connection. The developed real time tracker was designed for the shipping industry, where continuous monitoring for temperature (package content supervision) and acceleration (package integrity) is required [7,8]. The device was designed to have two months of autonomy and in this time interval, the data acquired are sent every hour to the client PC. Due to the high power consumption of GPRS, powering from a battery becomes a challenge in remote monitoring [9]. The proposed configuration is a generic device, which allows easily changing the amount and type of sensors in the system. In order to communicate with the sensors, Serial Peripheral Interface (SPI) and Inter-Integrated Circuit (I2C) interfaces are used. In the current design status, temperature and acceleration sensors are integrated. However, the device firmware and software architecture is designed to be user friendly, allowing the addition of new functionalities like humidity, pressure measurement or light detection. To guarantee worldwide communication coverage, a GSM quad-band module (850/900/1800/1900 MHz) was implemented on the device.

Comparing to other products on the market used in package tracking [10,11,12,13,14], the device is designed for harsh conditions (temperature range −30 °C to +80 °C) and a larger autonomy [10,11,12], (1 h reporting lasts up to 60 days). XpertTrack was designed to work below freezing point and for long periods of time, having a higher weight (*i.e.*, 850 g because of the large battery) than other devices. The important advantage is the high accuracy temperature measurement (±0.1 °C accuracy and ±0.01 °C sensitivity), which is usually found in high accuracy temperature data loggers or reference probes. The probe sensor can go over the device temperature range, supporting cryogenic temperatures (−200 °C) and very high temperatures (+200 °C) and it is connected through a flexible cable of 480 mm (customizable to 1000 mm).

When it comes to shock or free fall detection, XpertTrack measures acceleration on three axes. The sensor has 10 configurable levels in order to determine the shock intensity or if a free fall takes place. In through the implemented software, instant alerts up to 16 G can be triggered, which is similar with other products described in [10,11,12,13].

The XpertTrack was not designed for small packages, but more for large merchandises were weight is not an issue. The device’s accuracy is proved to be critical in cold chain shipments. The client is assured of the product temperature accuracy through a calibration certificate.

The system architecture has an Access Point, which allows transferring the data from the real time tracker to a PC much faster than using GSM connection. Compared to a USB connection [10,13], having a high speed wireless communication allows configuring and checking the sensor status without needing to open the container.

## 2. XpertTrack: Technical and General Specifications

**Figure 1:**
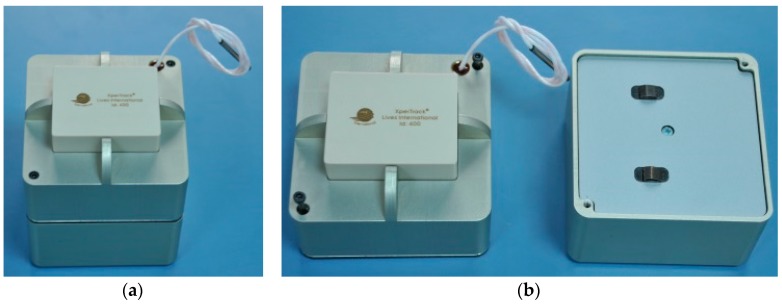
XpertTrack: (**a**) encapsulated device; and (**b**) electronic part and Batteries part.

XpertTrack, presented in Figure 1, was designed to be suitable for applications like: cold chain shipments, logistics, package integrity and remote monitoring. The alarms are triggered when products exceed the allowed temperature and vibration during shipments. In the case that the shipment has been tampered with, the current position and the temperature of the container are transmitted. XpertTrack technical and general specifications are mentioned in Table 1 and Table 2.

## 3. General Overview of System Architecture 

**Figure 2:**
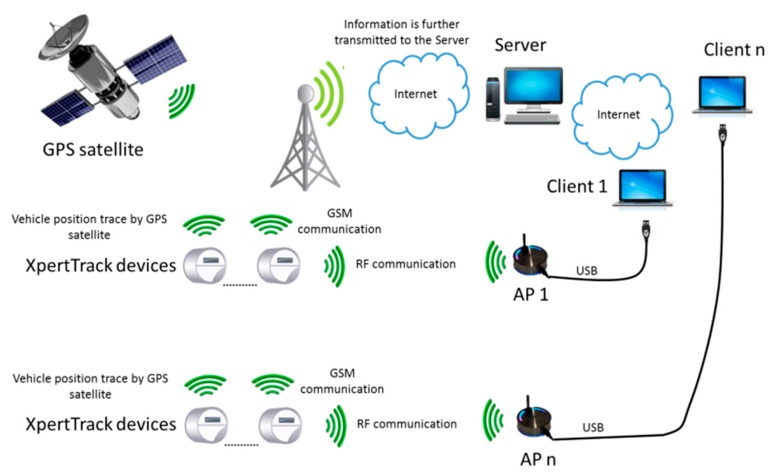
General system architecture.

XpertTrack does not only store the measurements (*i.e.*, temperature and acceleration), but also sends data over the Internet to a server using the GPRS protocol over GSM. Besides offering information regarding container integrity, the device is also capable of giving tracking data. Figure 2 presents the methods used to determine the cargo position: GPS satellite positioning or GSM triangulation. The GSM connection is not only used to transfer data between the devices and server, but also during configuration. 

**Figure 3:**
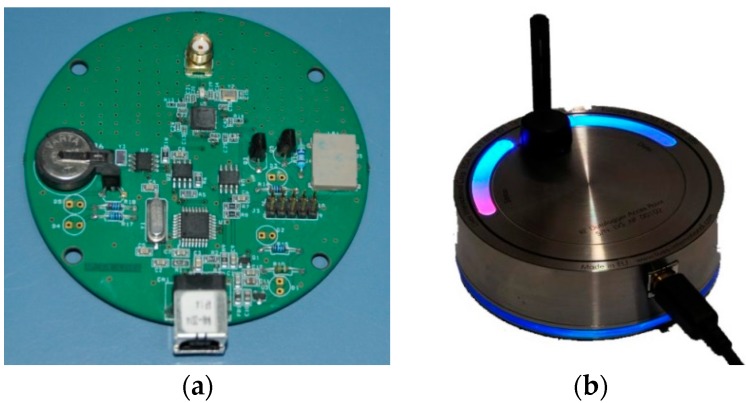
Access point: (**a**) printed circuit board, top side; and (**b**) encapsulated one.

In the case that a GSM connection is not available, a backup solution was designed involving a wireless connection to an Access Point unit (in a range of up to 200 m) connected to a client PC. Figure 3 presents the AP that converts the radio signal data packages into USB data and *vice-versa*, creating a two way communication between XpertTrack and the client PC. The AP has been created (designed) like in [15], based on the CC2530 microcontroller [16].

## 4. Device Concept

### 4.1. Electronic Design

The architecture is not straightforward, because of the high complexity of the entire system. Due to the necessity of a high number of peripherals, one microcontroller was not enough and a two-microcontroller architecture was needed. 

The main microcontroller is a low power one used in the peripheral communication and for commanding the slave microcontroller, which is responsible for GPS positioning, GSM positioning and GPRS communication.

**Figure 4:**
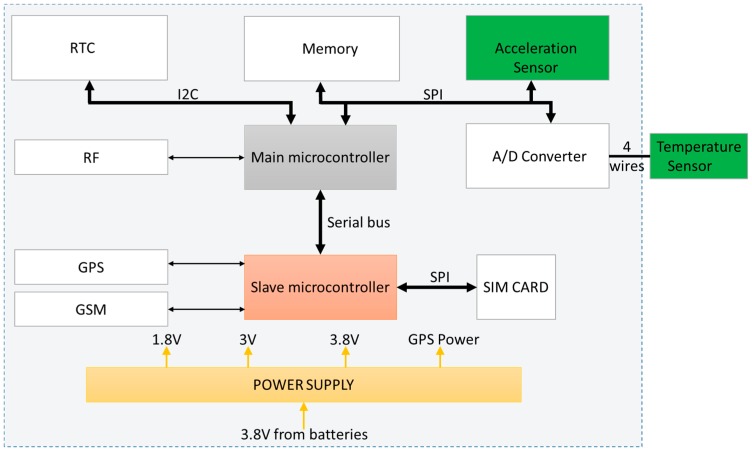
XpertTrack block structure.

Beside the two microcontrollers, an external Real Time Clock (RTC) was used for precise timing. The acquired data are saved in an external memory controlled by the main microcontroller. An analogue to digital converter for high precision temperature measurement and a low power acceleration sensor were also added. Figure 4 shows the block diagram.

For controlling the device, the main microcontroller is a CC2530 from Texas Instruments [16], with 256 KB of integrated Flash, which is enough for the software application. This microcontroller is used to acquire data from all sensors, to save the data and command the slave microcontroller. The CC2530 has an integrated IEEE 802.15.4 compliant radio frequency transceiver which is used to communicate in short ranges [16,17]. An AP has been created for the backup communication between the autonomous device and the client PC using the frequency of 2.4 GHz, the Industrial, Scientific and Medical band (ISM band).

The second microcontroller, the slave one, is a GE910-GNSS from Telit [18]. Comparing with other similar products, the microcontroller from Telit has lower power consumption. Other advantages are: embedded GPS receiver, working at low temperatures (*i.e.*, −30 °C and below), and relatively simple to use. The fact that it comes with an already-written GSM application software is a plus. Communication protocol (serial bus) with the TELIT module is achieved using AT commands, which are generic commands [19,20]. Using this type of communication ensures easy interchangeability with other similar microcontrollers.

Between the acceleration and temperature measurements, the most important one is the temperature reading. Because a high-end device was required, one of the best temperature sensors has been chosen: a PT100 resistive temperature sensor. This type of sensor can have different classes of precision, all errors being specified at 0 °C: Class C (±0.6 °C), Class B (±0.3 °C), Class A (±0.15 °C), 1/3 DIN (±0.08 °C) and 1/10 DIN (±0.03 °C). In the current design, 1/10 DIN precision class was chosen, with regards to achieving high measurement accuracy.

In order to benefit from the temperature sensor sensitivity, a high accuracy analogue to digital converter was also chosen. In the final design an AD7794 converter from Analog Devices was used [21]. This converter is a 24 bits Delta Sigma converter, with a built in an integrated current source for driving the measuring sensor. By doing so, the number of components, the measurement noise and the current consumption are reduced. Using a ratio measurement with a 1 KΩ high precision resistor guarantees best performance, as shown in the following equation:

(1) $$\mathrm{1LSB}=\frac{1K}{2^{24}-1}=5.96\times 10^{-5}$$

The AD7794 datasheet specifies 22 effective bits at a 240 ms conversion time which results in an effective measurement unit of 2.38 × 10^−4^ Ω. By converting this value into temperature measurement, a precision of 0.00061 °C is obtained, which is well below the accuracy of the PT. The data exchange between the slave microcontroller and the AD7794 is achieved through a SPI.

The high accuracy of the PT100 is not sufficient without a proper calibration, so all sensors are calibrated using ITS-90 (International Temperature Scale of 1990) calibration coefficients [22]. The standard offers calibration points in accordance with used temperature ranges and recommends calibration equations so that high performances can be obtained.

(2) $${W(T}_{90})={R(T}_{90})/R(273.16\;K)$$

(3) $$\Delta W={W(T}_{90})-W_{r}{(T}_{90})$$

Temperature is determined in Equation (2) by the ratio of the resistance R(T_90_) at temperature T_90_ and the resistance R(273.16 K) at the triple point of water [22]. T_90_ represents a mean of platinum thermometer resistance calibrated at specified sets of defining fixed points, and using specified reference and deviation functions for interpolation at inverting temperatures. In the specific thermometer resistance ranges, this parameter is obtain from W_r_(T_90_) given by the reference functions Equation (4) or (6) and the deviation ΔW Equation (3). The deviation is obtained directly from the calibration of the thermometer at intermediate temperatures, using means of the deviation function Equation (5) or (7). 

In the current implementation, the temperature range was split into two temperature sub ranges:

- For temperatures below 0 °C, Equation (4) is used, and the deviation ΔW is determined using relation Equation (5):
  (4) $$T_{90}/273.16K=\left(B_{0}+\sum_{i=1}^{15}B_{i}{\left(\frac{W_{r}^{\frac{1}{6}}-0.65}{0.35}\right)}^{i}\right)$$
  (5) $$\Delta W=a_{5}\left({W(T}_{90})-1\right)+b_{5}{\left({W(T}_{90})-1\right)}^{2}$$
- For temperatures above 0 °C, Equation (6) is used, and the deviation ΔW is determined using relation Equation (7):
  (6) $$T_{90}/K-273.15=D_{0}+\sum_{i=1}^{9}D_{i}{\left(\frac{W_{r}{(T}_{90})-2.64}{1.64}\right)}^{i}$$
  (7) $$\Delta W=a_{10}\left({W(T}_{90})-1\right)$$

Acceleration data are acquired using a LIS3DH from ST Microelectronics. This sensor is a digital one, so other data processing is not necessary. It can be set to measure acceleration, free fall, double click actions and many more. The main advantage of the selected sensor, in addition to its low power consumption, is the property of having interrupts on configurable thresholds. By doing so, battery saving can be achieved. The communication with the acceleration sensor is accomplished through an I^2^C interface.

The Real Time Clock (RTC) selected is an EM-3027, designed by EM Microelectronic. This circuit has encapsulated a typical current consumption of 0.8 μA, it is temperature compensated and it can be programmed as an alarm clock. This last functionality is used to wake-up the whole device from sleep state and to trigger the process based on the firmware architecture. The communication with the RTC is accomplished through Serial Peripheral Interface (SPI).

The collected data are saved in an external 16 Mbit flash, AT45DB161E, produced by Adesto. The low power consumption, small dimensions and extended temperature range makes it perfect for the intended application. By using a shared architecture, around 128,000 temperature samples, GPS position, GSM position, and 20,000 acceleration samples can be stored in the memory. The memory is accessed via Serial Peripheral Interface (SPI).

As was mentioned, a challenge of designing of XpertTrack was the power supply. The required lifetime was two months; therefore several Lithium-Ion batteries have been used. For the physical design, the power supply is represented by two sets of rechargeable batteries from ENIX, each having a capacity of 6.8 Ah, resulting in a total capacity of 13.6 Ah at 3.8 V. These two batteries guarantee that the device can be awaken every hour to make the transmission, during a period of 60 days.

### 4.2. Firmware Design

**Figure 5:**
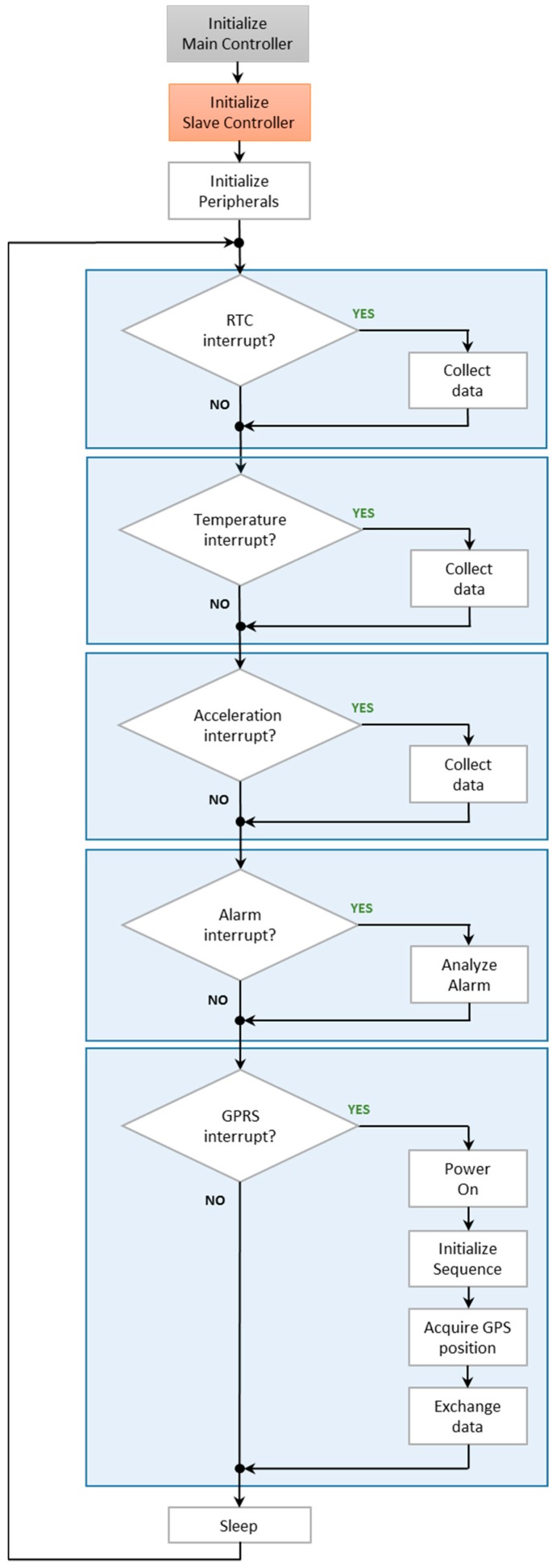
Firmware architecture.

Figure 5 shows the firmware architecture based on Round Robin, with interrupts and state machines.

First step is to initialize the main microcontroller, then the slave microcontroller, and in the end all other peripherals are initialized. After all initializations have finished successfully, the main loop is started. From this moment on, the main microcontroller will run the interrupt routines, which are triggered by the peripherals. In the awake state, initiate by the RTC alarm, the temperature and all other parameters values are acquired. The new data are stored in the memory chip.

The TELIT module is configured by the main microcontroller and its functionality consists in the following steps: power-up, search for signal, connect to GPRS and acquire GPS and GSM positioning. After the initialization is finished successfully, the exchange of information with the server starts.

On top of basic functionality, an alarm system is implemented. The final user (client) can define multiple alarms on the sensors (*i.e.*, temperature and acceleration) and a handling routine. If the temperature or acceleration is not within the defined limits, the device handles the information as the user defines it: send data at different time intervals, stream temperature/acceleration values, *etc.*

The messages used to exchange data between XpertTrack and the server has the format explained in Table 3.

Each message contains 1 byte that specifies the length of the message, 1 byte is used to specify the device’s type, 2 bytes identifies the device’s address, 2 bytes are used for the command code, the parameters are optional and, if present, have variable length, 2 bytes are used for information integrity (CRC16 is used), and the last 1 byte is the end of message.

The messages are sent to a server specified by an URL (Uniform Resource Locator) or by an IP (Internet Protocol) address. The connection with the server can be performed on a specific port, for example HTTP (Hypertext Transfer Protocol) standard port (*i.e.*, 80), or a custom one, for security reasons. When an alternative random port is used instead of the standard HTTP port a supplementary level of protection against unwanted access can be achieved. The protection consists in an unknown port and the access is granted using user and password credentials (each XpertTrack has a unique user and a password generated using a proprietary algorithm). 

In order to be sure that the sent data reaches the destination, the communication is done using HTTP protocol, even if port 80 is not used. On top of that, the server must acknowledge the received information. Through this implementation, the communication concept guarantees that the information reaches the server and is analyzed.

Additional attention must be paid on power supply supervision. The slave microcontroller is not able to run below 3.4 V, whereas the rest of the components run at 3 V or less. The microcontroller’s internal A/D converter is used to measure battery voltage. In the case of low battery levels, XpertTrack is designed to stop the GPRS communication and continue to acquire and save the data. In this way, no data are lost and it can be recovered at destination. The server is notified with respect to the battery critical level, prior of communication interruption.

## 5. Device Implementation

The design and implementation of the XpertTrack was challenging: Radio Frequency (RF) antenna impedance tuning, GSM antenna impedance tuning, GPS antenna impedance tuning, power supply interference and several high speed communication lines [8,23,24]. Due to all these challenges, a four-layer design was chosen. This guaranteed easy decoupling, proper shielding between power planes and high speed communication lines, and relaxed routing. It provides easy decoupling of the power plane placed between the ground plane and the bottom layer, which is predominantly ground.

The maximum power theorem states that maximum power is transferred when the internal resistance of the source equals the resistance of the load. In order to obtain the complete impedance matching for antennas (RF, GSM and GPS) (50 Ω out/50 Ω line/50 Ω in), a microstrip design technology was implemented [17].

To keep the hardware dimensions as small as possible, for the radio frequency antenna a special RF meandered IFA (Inverted F Antenna) was used [17]. By doing so, the antenna dimensions are reduced to 15.2 mm × 5.7 mm. On the other hand, this method ensures a Voltage Standing Wave Ratio (VSWR) of less than 2 across the 2.4 GHz ISM band when the antenna is connected to a 50 Ω source.

### 5.1. Printed Circuit Board (PCB) Designing

**Figure 6:**
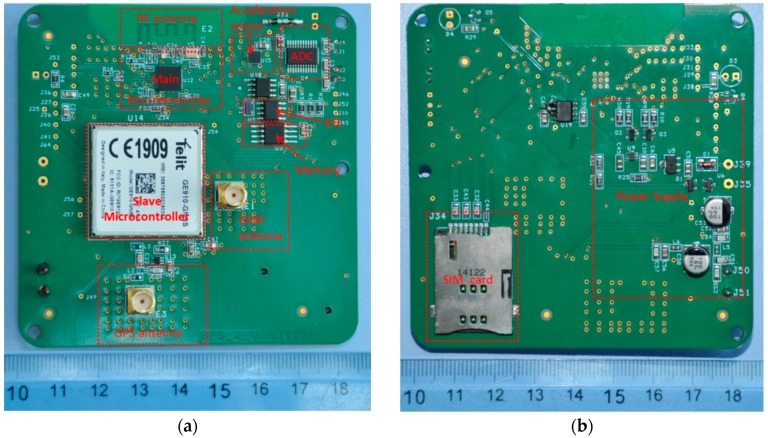
Printed Circuit Board (PCB) of the device: (**a**) Top Layer view; and (**b**) Bottom Layer view.

On the Top (Figure 6a) and Bottom planes (Figure 6b), the unused area was flooded with ground areas. Then these ground regions were connected to the ground plane. By doing so, the impedance seen by the returning currents for the RF, GSM and GPS modules is kept at the desired value. Considering the continuous ground plane underneath the design, all the connection to ground could be kept short and close to the integrated circuits. This approach avoids creating long traces that can add unwanted inductance to the connections, which decrease the performances.

### 5.2. Stack-Up Layers Definition

The PCB dielectric has to withstand temperatures between −30 °C to +80 °C and to resist to chemical attacks. Because of these requirements, IS410 was chosen as a dielectric. IS410 is based on a high-Tg epoxy system with a nominal glass transition temperature of +180 °C and is particularly well-suited for lead-free soldering processes, which subject materials to increasingly greater thermal stresses. The special properties for this material are:

- high thermal resistance; T260 > 60 min, T288 = 30 min, TD: +360 °C;
- high resistance to chemical attack;
- CAF-enhanced;
- excellent resistance to heat shock (withstands six solder test repetitions of 10 s at +288 °C); and
- completely cures without follow-up post baking.

**Figure 7:**
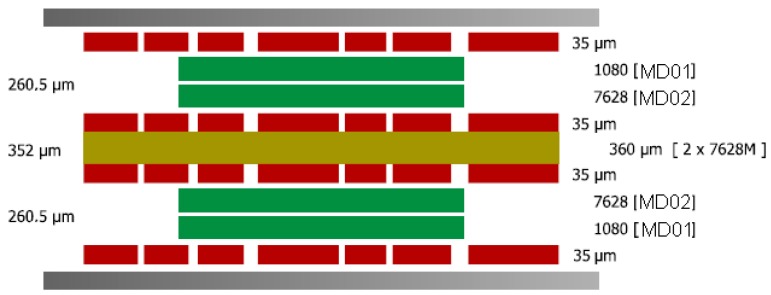
Stack-up layers definition for PCB.

The stack-up layers definition was made according to IPC-4562 and based on the material proprieties, as presented in Figure 7. 

### 5.3. Software Details

**Figure 8:**
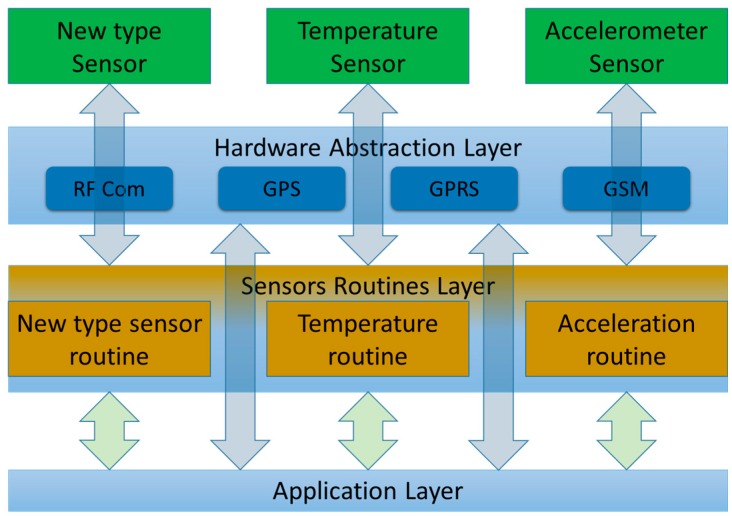
The Firmware includes a certain number of modules working.

Due to its design, the microcontroller firmware can easily be modified to support new communications protocols or new types of sensors (see Figure 8). At the lowest level is the Hardware Abstraction Layer (HAL), where all communication protocols have been implemented. The code is split into modules, each one being responsible for one component. Each module calls HAL functions, processes the data and sends them (when requested) to the main program. Any modification to the design implies adding, changing or removing one or more modules.

The server software is built on a Java platform and runs on a dedicated computer with a public IP. However, in the near future, Webhost features will be implemented on the server. All the exchanged data are being stored on an SQL Database with several layers of protection. In addition to the Java platform, different software (called client software) has been developed to communicate with the server, extract and display the required information and to ensure surveillance on the threshold levels. With this configuration, all data can be seen from all over the world at any given moment. Even when data cannot be sent to the server due to connection problems, the acquired samples from the sensors are not lost, but stored in the device internal memory, based on a FIFO stack-up. The information can be recovered, either when the connection is re-established, or by downloading it by the user through the wireless connection using the AP.

### 5.4. Programming and Configuring the Device

The programming is done by the client through the software. The user can set the scan rate (at one hour, XpertTrack can run for more than two months), the update rate, the shock sensitivity and several alarms. After this, the software checks for GSM signal and when it gets it, sends all the information. From this point on, the device is responsible for data transmission and proper functioning. However, the user can modify certain parameters and will be notified when requested changes have been accepted.

### 5.5. Data Reports

The information is seen by the user in Graphs, Reports, Statistics, and most important: points on the map. This helps to monitor where the shipment has travelled and how were the conditions inside the cargo.

## 6. Experiments and Results

### 6.1. Communications Tests

#### 6.1.1. GPS and GSM

First sets of tests were performed at room temperature to check the communication reliability and quality. Positive tests have been performed measuring QoS (Quality of Service) of the GSM and GPS link. Due to the impedance matching, the device managed to get GSM signal everywhere a regular cell phone would work. In near future, tests will be done in areas with poor mobile phone coverage.

Measurements to verify GPS accuracy have been made and were determined to have an accuracy of 10 m or less even if the receiver is in a building. If due to exceptional situations, GPS signal is not available, the location is determined using GSM triangulation. An algorithm based on signal strength and BTS (Base Transceiver Station) positions has been implemented, which resulted in positioning error ranging from 50 m (if the device is located in a city with lots of antennas) to a few kilometers (if the device is located in a region with only one or two antennas).

A worst case testing has been performed: where during startup or in the middle of the communication, the GSM antenna has been removed causing communication lost. When that happened, a lack of GSM signal was detected and the communication dropped. At the next regular wake-up, the communication has been re-established and the data have been transmitted from the moment when the communication was dropped.

The communication quality was tested in harsh temperature ranging from −30 °C to +80 °C [25]. The communication between the two microcontrollers was monitored for 24 h and no errors were detected. Although the device was in a freezer at −30 °C or in an oven at +80 °C, the data arrived to the server without any loses.

**Figure 9:**
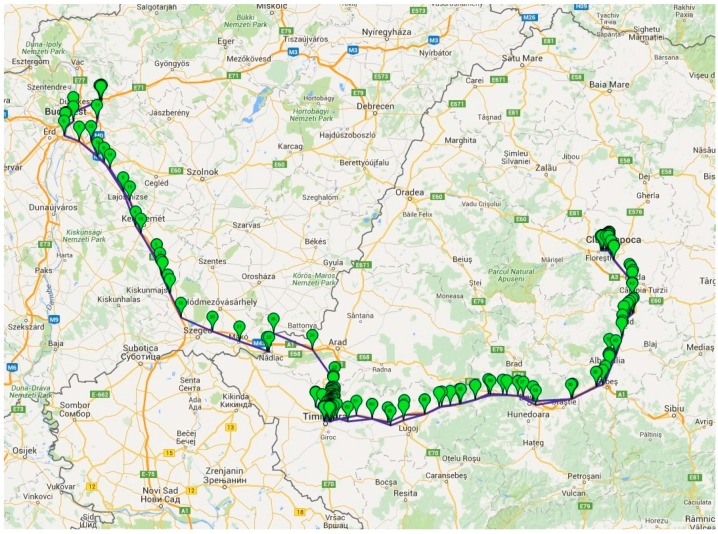
Results of GSM positioning for travelling from Romania to Hungary.

**Figure 10:**
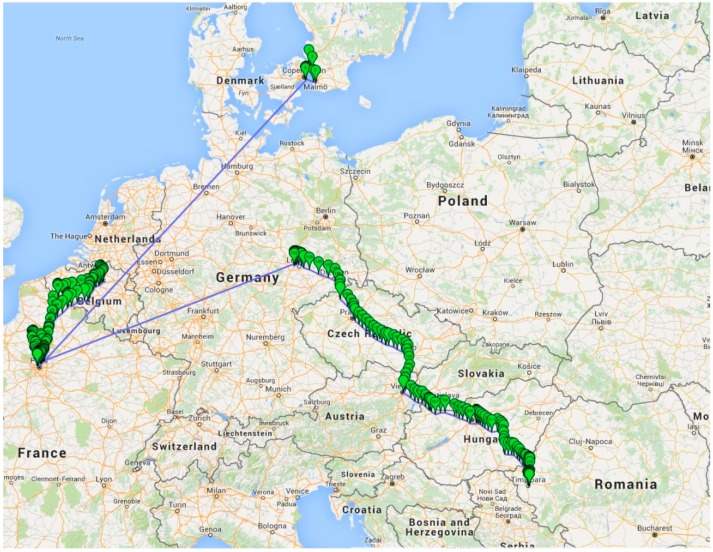
Results of GSM positioning for travelling from Romania to Denmark.

Besides testing in a controlled environment, real live testing was also done. Figure 9 and Figure 10 shows the test results of GSM positioning. As the GPS coordinates from the Figure 10 show, even if the connection is lost due to travelling by airplane (the long lines from Germany to France, and from France to Denmark), the transfer of data is re-established when GSM signal is redetected. Considering that the GSM penetration is far beyond the GPSs, especially if the XpertTrack is in a package in a metal container, it can be tracked almost everywhere on the globe.

The main application of the device is to supervise the temperature change (which include alarms, *etc.*) and secondly the position. This leads to the following scenario: once the position is acquired, it is not acquired anymore for a user defined time interval. In Figure 9, the spatial gap between GPS marks is big, because the device was in a package that was in a moving car, running at 130 km/h on a highway. We considered this to be the worst case scenario and we found out that the GSM signal is lost very often, which turns into a huge battery consumption, due to module signal searching (consumption of hundreds of mA). This case would drain the battery and that is inadmissible, therefore the decision of temperature constant supervision and range tracking has been taken.

Communication protocol is based on TCP/IP, so no packages are lost. Confirmation of the sent or received packages is used at each end. This guarantees that all desired data are sent. If something unexpected occurs, like sudden loss of signal, affecting the exchange of information, the communication is dropped until GSM signal is received once again.

#### 6.1.2. Radio

Radio quality has been tested at room temperatures and package loss was found to be <1% in normal conditions. The performance was achieved by developing a firmware that implements the data retransmission and confirmation for each package. Tests have also been conducted to check Line of Sight performance and communication have been present even at 200 m between XpertTrack and the AP.

### 6.2. Temperature Measurements

The temperature measurement has been subjected to intensive testing, starting at −80 °C and finishing at +140 °C. After the functional testing, calibration has been performed successfully. Further tests have been made, starting with −80 °C, −40 °C, 0 °C, +60 °C and finishing at +140 °C.

**Figure 11:**
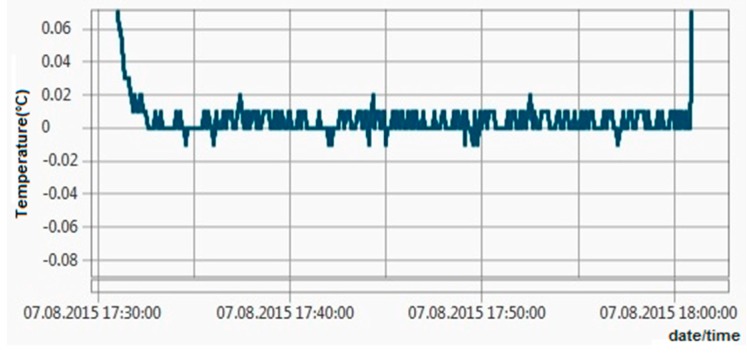
Experimental measurements in an oil bath at 0 °C.

**Figure 12:**
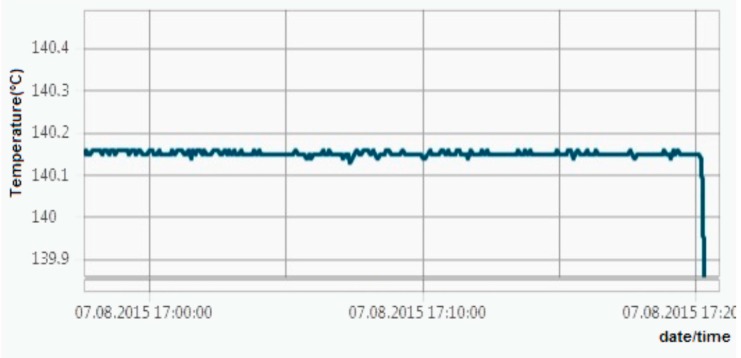
Experimental measurements in an oil bath at +140 °C.

**Figure 13:**
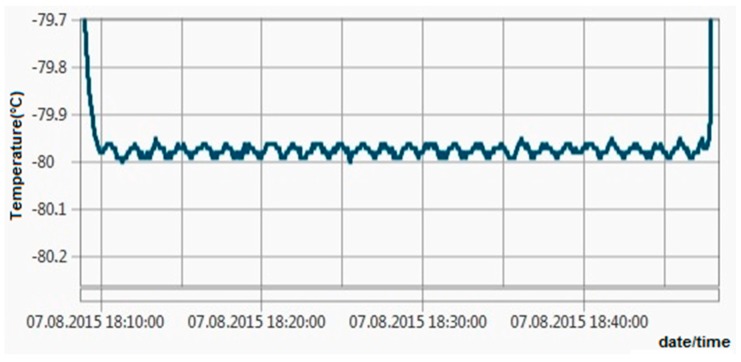
Experimental measurements in an oil bath at −80 °C.

**Figure 14:**
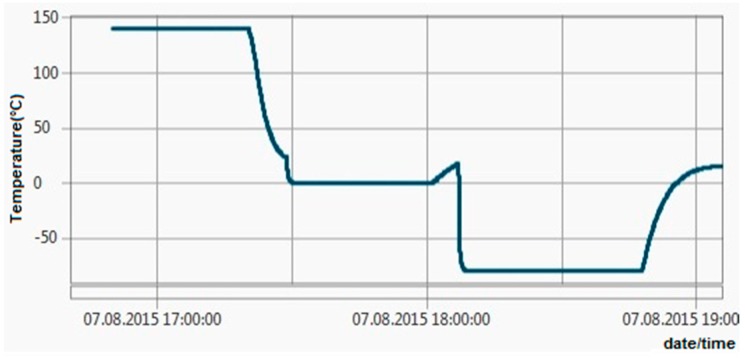
Experimental measurements, in an oil bath, to test the temperature response from +140 °C to −80 °C.

All these tests revealed maximum errors of ±0.06 °C, so the ±0.1 °C requirement has been achieved. Figure 11, Figure 12, Figure 13 and Figure 14 show experimental measurements in an oil bath. As the measurements show, a very low ripple (less than 0.01 °C) was achieved, ensuring once again a very high precision.

**Figure 15:**
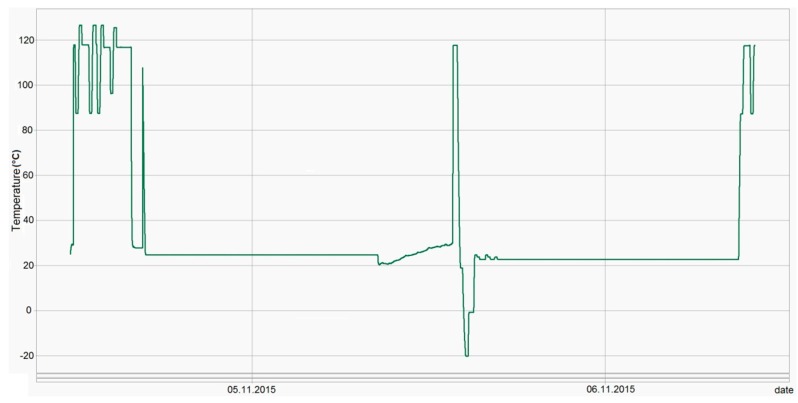
Experimental measurements in professional temperature calibrator (Temperature Dry Block PTC-155 Ametek).

In Figure 15, different temperature test patterns done in Temperature Dry Block PTC-155 from Ametek can be observed. During these tests, the response time and measurement stability were tested. 

First tests consisted in measuring cycles of temperature values between 90 °C and 120 °C. To test the stability, the same cycles were repeated.

The next step was to take a measurement cycle for 23 °C and raise the temperature during the cycle in order to test the response time. Further, the stability was tested at 23 °C and again at 120 °C.

Additionally, the stability was tested also for negative temperatures (*i.e.*, *−*20 °C) for a measurement cycle and the response time for switching temperature was determined. All the temperature measurements were done relative to a temperature reference probe.

**Figure 16:**
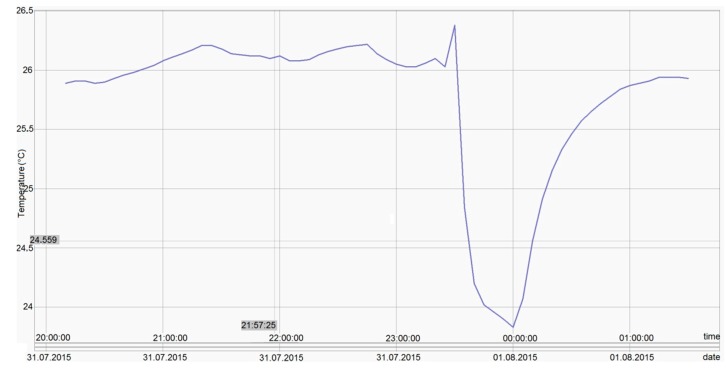
Device temperature measurements during merchandise monitoring.

The tests results obtained in the temperature calibrator were also confirmed during on site exploitation (*i.e.*, merchandise shipment, see Figure 16). 

### 6.3. Shock

Like the temperature measurements, the shock monitoring also has high precision. The accelerometer sensor allows four configurable sensitivities (1, 2, 4 or 12 mg/digit), depending on the type of application in which it is used. Testing was also done on the accelerometer sensor, during which it was configured for different sensitivity levels. 

### 6.4. Power Management 

Another very important parameter is the battery life [26]. The measurements revealed a sleep power consumption of 300 μA and an average of 200 mA during GPRS communication. The measurements revealed an average of 87 s from power-up to sleep, which resulted in an average current of 4.83 mA over an hour. If the two values are added, the average consumption is 5.13 mA over an hour. The batteries have a total capacity of 13.6 Ah, which at the average current consumption resulted in a theoretical autonomy of 2651 h (about 110 days). Considering that a typical battery during working conditions usually has only 70% of its stated maximum capacity, a 77-day working period results. Currently, XpertTrack is in commercial exploitation for about one and a half months without stopping or reporting faulty operation.

## 7. Conclusions and Future Works

This paper presents a generic device that can be used in monitoring temperature and shock values with the important advantage of being autonomous. The core is created and the implementation of any other feature (e.g., new type of measurements) can be done without major changes to the device. At this moment, the proposed solution is based on a high accuracy temperature sensor, an accelerometer sensor (for shock detection), a GPS receiver and a GSM data transfer (through GPRS) and positioning. Besides the previous features, a wireless connection was also implemented in order to facilitate an easy remote access and configuration to the device during exploitation. Regarding the price, for now the device has been constructed in prototype costs and marketing research is still under the process, so the market price of the product is not known yet. The production volume of the device is a critical aspect concerning the price.

As future development, a more power efficient GSM/GPS module could be used, in order to achieve an increase in autonomy. For the use of the device in merchandise shipments by airplane, a special sleep mode accepted by aeronautic EMC standards shall be developed.

## Figures and Tables

**Table 1:** Technical specifications.

Characteristics	Temperature	Shock
Sensor type	4 wires PT100	acceleration
flexible	PTFE 19“ (480 mm)	
customized	Up to 39“ (1000 mm)	
Range	−30 °C to +80 °C	
	(−200 °C to +200 °C sensor only)	
Accuracy	±0.1 °C	
Sensitivity	±0.01 °C	10 levels (shock/free fall)

**Table 2:** General specifications.

Characteristics	Details
Weight	850 g battery included
Antennas Battery	internal 2 lithium batteries
Battery lifetime	Up to 3 month—based on user (client) usage
Size	90 mm × 90 mm × 100 mm
Sampling rate	2 s to 24 h
Logger update rate	300 s to 24 h—user configurable
Memory capacity	128.000 data points
Internal clock drift	4 s/24 h @ +23 °C
Calibrations	Factory: Traceable NIST/COFRAC—IST-90 coefficient reside in internal memory
	User: Close loop calibration, on user site, using provided software for devices

**Table 3:** Messages format.

Length	Device Type	Address	Cmd.	Param.	CRC	End
1 byte	1 byte	2 bytes	2 bytes	0…n byte(s)	2 bytes	1 byte
